# New *Cernotina* caddisflies from the Ecuadorian Amazon (Trichoptera: Polycentropodidae)

**DOI:** 10.7717/peerj.3960

**Published:** 2017-10-27

**Authors:** Lucas M. Camargos, Blanca Ríos-Touma, Ralph W. Holzenthal

**Affiliations:** 1 Department of Entomology, University of Minnesota—Twin Cities Campus, St. Paul, MN, United States of America; 2 Facultad de Ingenierías y Ciencias Agropecuarias, Ingeniería Ambiental, Grupo de Investigación en Biodiversidad, Medio Ambiente y Salud -BIOMAS-, Universidad de las Américas, Quito, Ecuador

**Keywords:** Aquatic macroinvertebrates, Homology, Species description, Endemism, Taxonomy, Morphology, Amazonian streams, Neotropics

## Abstract

Two new species of the caddisfly genus *Cernotina* Ross, 1938 (Polycentropodidae) are described from the lowland Amazon basin of Ecuador, *Cernotina tiputini*, new species, and *Cernotina waorani*, new species. These represent the first new species described from this region. We also record from Ecuador for the first time *Cernotina hastilis* Flint, previously known from Tobago, and present new Ecuadorian locality records for *C*. *cygnea* Flint, and *C*. *lobisomem* Santos & Nessimian. The homology of the intermediate appendage of the male genitalia of this genus is established. The region surveyed is under severe environmental threat from logging, mining, and crude oil extraction, making the description of the biodiversity of the region imperative.

## Introduction

Trichoptera are an order of insects found in all faunal regions and is comprised of almost 16,000 described species. It is the largest insect order in which all included species live in freshwater during the immature stages (except for a very few semi-terrestrial species and even fewer marine species) (Holzenthal, Thomson & Ríos-Touma, 2015). The Neotropical region (Mexico, Central America, the Caribbean, and South America) is especially diverse in Trichoptera, with more than 3,200 species currently known (Holzenthal & Calor, 2017). Because of their high sensitivity to pollution and environmental changes, caddisflies are considered to be biological indicators of the quality of freshwater (Chang et al., 2014). Various biological indices and metrics have been developed incorporating caddisfly diversity and abundance to assess and monitor water quality by many national agencies around the world, including those in South America (Ríos-Touma, Acosta & Prat, 2014).

Among the 39 extant families of Trichoptera, the cosmopolitan family Polycentropodidae contains about 650 species and 15 genera (Chamorro & Holzenthal, 2011). Five genera of polycentropodids occur in the Neotropics: *Cernotina* Ross, 1938, *Cyrnellus* Banks, 1913, *Nyctiophylax* Brauer, 1865, *Polycentropus* Curtis, 1835, and *Polyplectropus* Ulmer, 1905 (Holzenthal & Calor, 2017).

As an exclusively New World genus, *Cernotina* has most of its 70 extant species in the Neotropical region (Holzenthal & Calor, 2017), where most occur in the lowlands of the vast Amazon basin (Flint Jr, 1971). One species, *Cernotina pulchra* Wichard, 2007, is known from Dominican amber. No species occur in temperate southern South America (Chile and adjacent patagonian Argentina). In the central and northern Andean countries, *Cernotina* is found exclusively in the Amazonian lowlands (Holzenthal & Calor, 2017). In spite of its diversity and apparently wide distribution, published descriptions and records from South America are few and include those from Argentina (Flint Jr, 1983), Brazil (Flint Jr, 1971; Flint Jr, 1991; Holzenthal & Almeida, 2003; Santos & Nessimian, 2008; Dumas & Nessimian, 2011; Barcelos-Silva et al., 2013), Peru (Sykora, 1998), and Uruguay (Angrisano, 1994). The first records of the genus from Ecuador were recently published for *Cernotina cygnea* Flint Jr, 1971 and *C*. *lobisomem* Santos & Nessimian, 2008 (Ríos-Touma et al., 2017). In North America, *Cernotina* inhabits lotic and lentic freshwaters habitats and the larvae are considered predators (Morse & Holzenthal, 2008). However, there is no ecological information for the Neotropical species.

Polycentropodidae can be distinguished from other Neotropical caddisflies by a combination of characters (Chamorro & Holzenthal, 2011; Pes et al., 2014): absence of ocelli; elongate, flexible segment 5 of the maxillary palp; segment 3 of the maxillary palp inserted subapically on segment 2; pair of distinct, oval, setal warts on the mesoscutum. Adult *Cernotina* can be separated from other Neotropical polycentropodids by the absence of a preapical tibial spur on the foreleg.

The eastern part of the Ecuadorian Amazon includes the vast Yasuní National Park (ca. 10,000 km^2^) and the adjacent, much smaller and private Tiputini Biodiversity Station (6.5 km^2^). These conservation areas harbor a great diversity of amphibians, mammals, birds, and plants (Bass et al., 2010). In contrast, most insects, including Trichoptera have not been intensively studied in this area. However, while existing records are scarce, they suggest a diverse fauna (Ríos-Touma et al., 2017). Oil concessions and logging have been threatening the biological diversity of this region for more than five decades (Bass et al., 2010; Sierra, 2000; Viña, Echavarria & Rundquist, 2004; O’Rourke & Connolly, 2003). Further, since freshwater biodiversity is among the world’s least known (Dudgeon et al., 2006; Esteban & Finlay, 2010), and the Amazon is among the greatest global freshwater ecosystems, it is imperative to study well preserved areas like Yasuní and Tiputini. In our recent effort to record species of Trichoptera from Tiputini, we found new species and records of *Cernotina* (Ríos-Touma et al., 2017) among other caddisflies. In this paper, we describe two new species of *Cernotina* from Tiputini and record a previously described species for the first time in Ecuador.

## Materials and Methods

**Figure 1:**
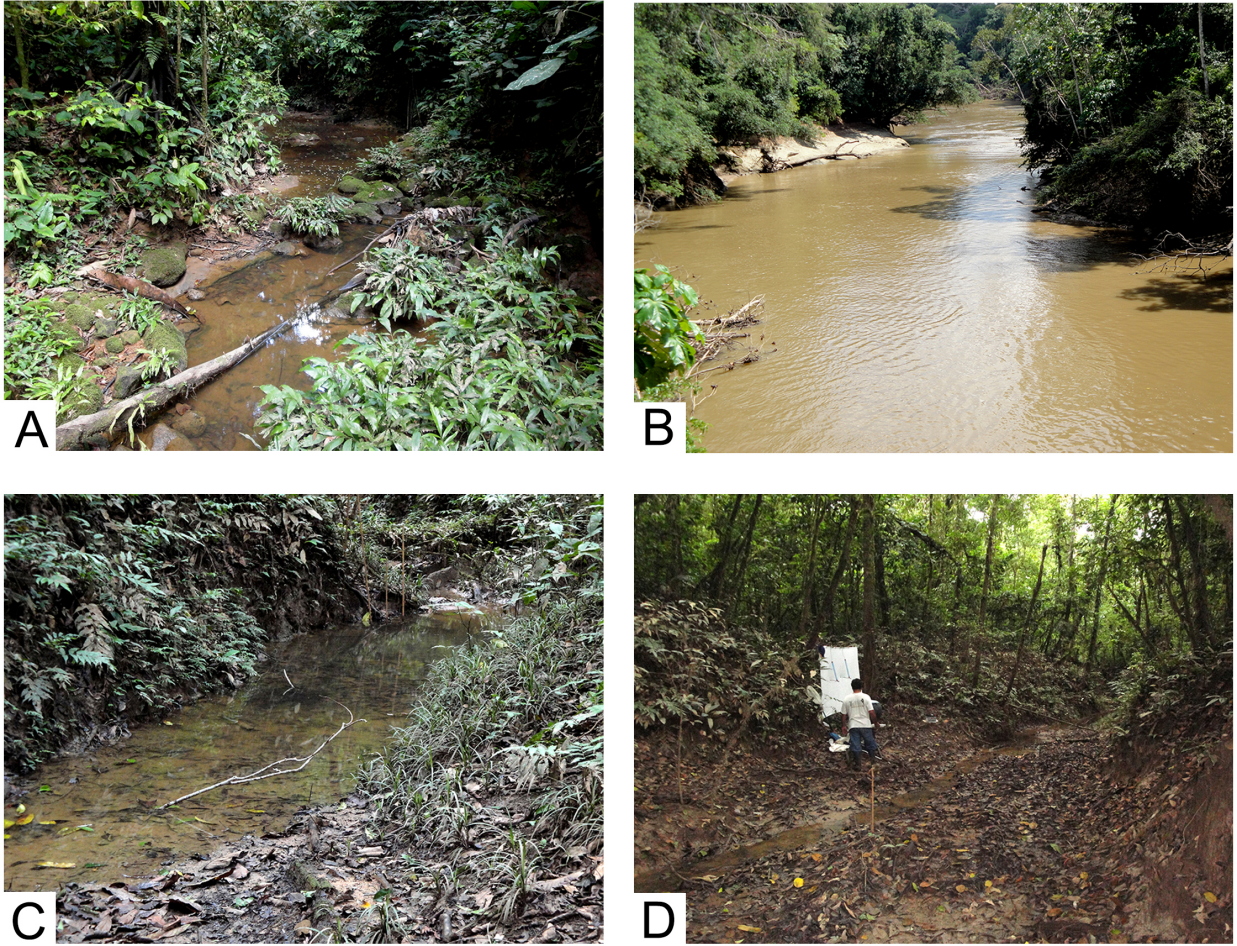
Collecting localities, Tiputini Biodiversity Station, Ecuador. (A) Small stream, Harpia trail, type locality for *Cernotina waorani*, new species. (B) Río Tiputini. (C) River slough, Numa trail, type locality for *Cernotina tiputini*, new species. (D) Same, showing UV light collecting method. Photographs by Ralph W. Holzenthal.

Collecting was accomplished at three sites in the Tiputini Biodiversity Station in October, 2011. The station is located on the northern bank of the Río Tiputini, an easterly flowing southern tributary of the much larger Río Napo (File S1). We sampled two small waterways and the Tiputini river using ultraviolet lights for approximately 2.5 h (17:30–20:00 h) (Fig. 1). To collect dry specimens for subsequent pinning, ultraviolet and white fluorescent lights were hung in front of a white bed sheet placed by the margin of the streams (Fig. 1D). Adult Trichoptera attracted to the lights were captured in jars containing ammonium carbonate as the killing agent. In addition, a small UV light was placed over a white tray containing 80% ethanol and left for about 2.5 h at streamside. Caddisflies collected in the tray were sorted later in the laboratory from other insects and were stored in 80% ethanol.

For examination and description, the male genitalia were prepared using warm 85% lactic acid to macerate soft tissue following the procedures of Blahnik, Holzenthal & Prather (2007). Pencil sketches were rendered with the aid of a drawing tube attached to an Olympus BX 41 compound microscope. Pencil sketches were imported into Adobe Illustrator CC to produce final digital illustrations. Terminology for male genitalic structures follows that of Chamorro & Holzenthal (2011) for Polycentropodidae.

The specimens examined in this work are deposited in the University of Minnesota Insect Collection, St. Paul, Minnesota, USA (UMSP), the Museo Ecuatoriano de Ciencias Naturales, Quito, Ecuador (MECN), and the Museo de Ecología Acuática de la Universidad San Francisco de Quito, Ecuador (USFQ) as indicated below. All collections were performed under the Environmental Ministry of Ecuador study permit No 0032 MAE-DPO-PNY-2011.

The electronic version of this article in Portable Document Format (PDF) will represent a published work according to the International Commission on Zoological Nomenclature (ICZN), and hence the new names contained in the electronic version are effectively published under that Code from the electronic edition alone. This published work and the nomenclatural acts it contains have been registered in ZooBank, the online registration system for the ICZN. The ZooBank LSIDs (Life Science Identifiers) can be resolved and the associated information viewed through any standard web browser by appending the LSID to the prefix http://zoobank.org/. The LSID for this publication is: urn:lsid:zoobank.org:pub:5CE7AFEF-5077-4930-96BA-5B746FF12250. The online version of this work is archived and available from the following digital repositories: PeerJ, PubMed Central and CLOCKSS.

## Results

### Species descriptions

**Table:** 

**Figure 2:**
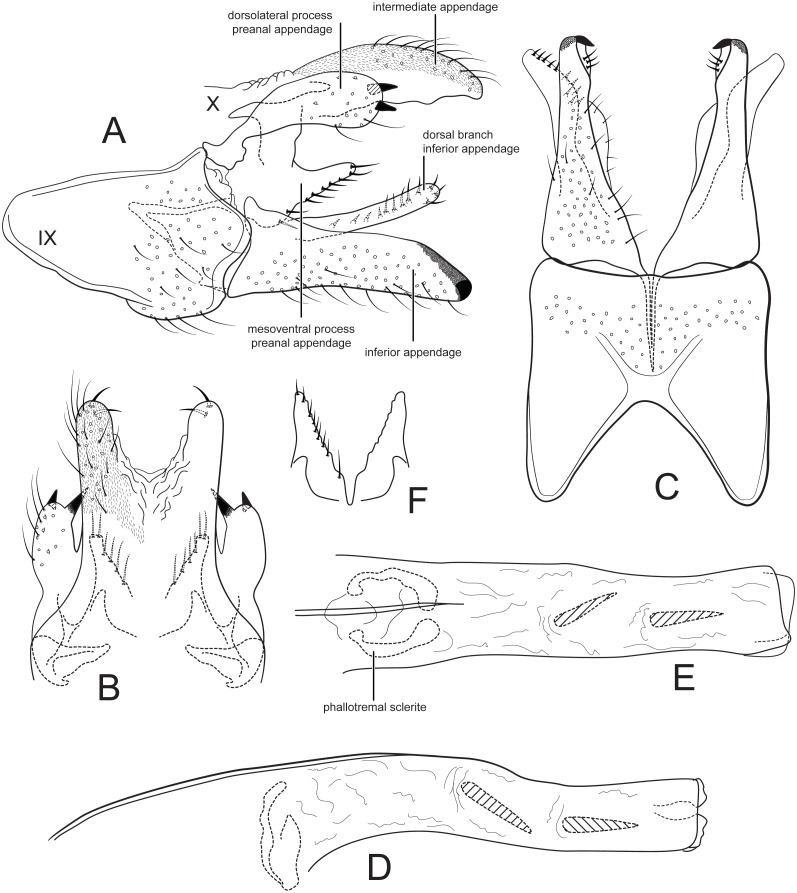
Male genitalia of *Cernotina tiputini*, new species. (A) Segment IX and X, lateral. (B) Segment X and preanal appendages, dorsal. (C) Segment IX and inferior appendages, ventral. (D) Phallus, lateral. (E) Phallus, dorsal. (F) Mesoventral processes of preanal appendages, ventral.

***Cernotina tiputini, new species***
urn:lsid:zoobank.org:act:E254D21B-7FA0-47CA-AF34-BE437CEE71CE
Figure 2.

This species is very similar to *C chelifera* Flint Jr, 1972 from Argentina in the two apical spines of the dorsolateral process of the preanal appendage and the general shape of the appendage. It differs from the Argentinian species by the overall shape of tergum X and the intermediate appendage, its relative size shorter than the inferior appendage, a broader dorsolateral process in dorsal aspect, a narrower inferior appendage, and by having two internal spines instead of only one long spine in the phallus.

Forewing length 3.5 mm male (*n* = 2). Forewing very light brown, apex with small patch of dark setae, white hairs along anal margin; head and thorax with white hair dorsally; antennae stramineous. Forewing with fork V petiolate; hind wing with cross vein Cu2–1A absent, vein 3A absent.

*Male genitalia*: Sternum IX with height 3/4ths of entire male genital complex, quadrate, anteroventral margin with deep, broad concavity. Tergum X semi-membranous, divided mid-dorsally; intermediate appendages slightly curved ventrad, thumb-like, about as long as inferior appendage, setose, with two thick apical setae, surface with microsetae. Preanal appendages each composed of two processes; dorsolateral process oblong, shorter than inferior appendage, with two apical spines; mesoventral process produced dorsolaterally, fused on midline, shorter than inferior appendage, bearing a row of stout setae on posterior margin. Inferior appendages in lateral view slightly fusiform, straight, apex rounded; sclerotized apicomesally, pointed in ventral view; dorsal branch elongate, about as long as body of appendage, oriented posteriad, bearing a row of setae. Phallus slightly bent at mid-length, narrow, with two spines; phallotremal sclerite large, ovate, with two apparent lateral processes.

***Holotype male:*** **ECUADOR:** Orellana, Reserva de Biodiversidad Tiputini, river slough, Numa trail, 00.63954°S, 76.14836°W, el. 260 m, 23.x.2011, Holzenthal and Ríos [pinned] (UMSP000098447) (UMSP).

***Paratype:*** same as holotype, except: one male [alcohol] (MECN).

**Etymology:** The species is named for the Tiputini River and the adjacent biodiversity research station.

**Table:** 

**Figure 3:**
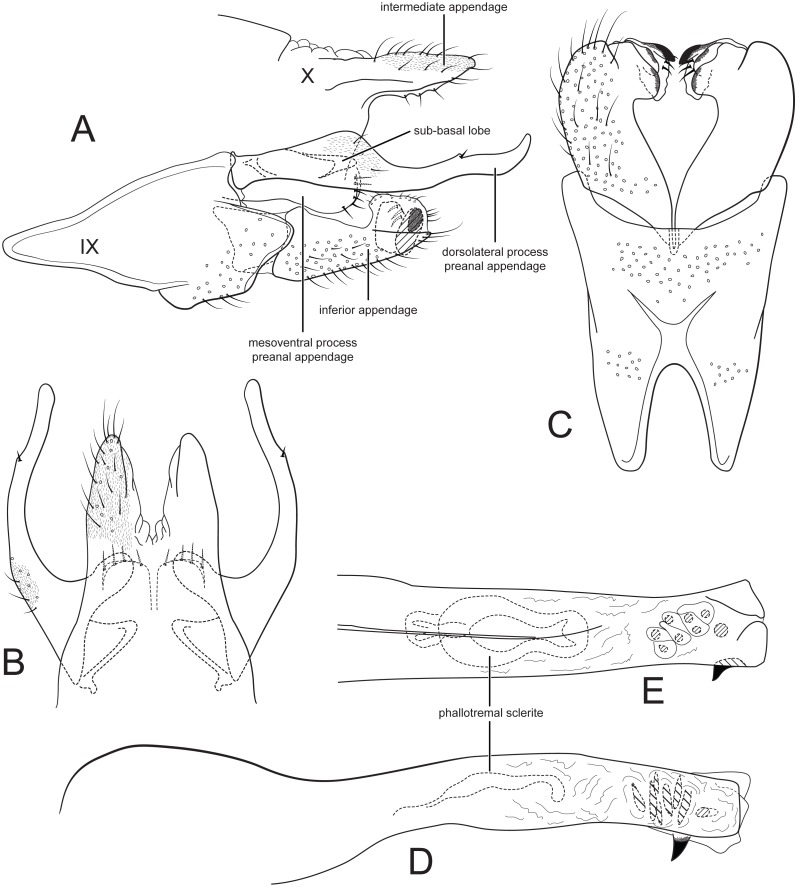
Male genitalia of *Cernotina waorani*, new species. (A) Segment IX and X, lateral. (B) Segment X and preanal appendages, dorsal. (C) Segment IX and inferior appendages, ventral. (D) Phallus, lateral. (E) Phallus, dorsal.

***Cernotinawaorani*, new species**
urn:lsid:zoobank.org:act:15FD59A3-69F2-4152-B7B8-EE5E34051603
Figure 3.

This species has similarities with *C*. *fallaciosa* Flint Jr, 1983 from Argentina in the bulbous apex of the inferior appendage in lateral aspect and the presence of multiple internal spines in the phallus. However, the absence of apical spines on the dorsolateral process of the preanal appendage, its shape, and the presence of a flap-like median, sub-basal lobe renders this species distinct.

Forewing length 3.5–4 mm (*n* = 6). Forewing stramineous, with slightly darker hairs at apex; head and thorax with lighter hair dorsally; antennae stramineous. Forewing with fork V sessile; hind wing with crossvein Cu2–1A present, vein 3A absent.

*Male genitalia*: Sternum IX with height about half of entire male genital complex, trapezoidal; anteroventral margin with deep, narrow concavity. Tergum X semi-membranous, divided mid-dorsally; intermediate appendages entire, digitate, about as long as inferior appendage, setose, without spines, surface with microsetae. Preanal appendages each composed of two processes: dorsolateral process elongate, longer than inferior appendage, with flap-like sub-basal lobe; mesoventral process oblong, not fused on midline, shorter than inferior appendage, bearing a row of stout setae on posterior margin. Inferior appendage subtriangular in lateral view, lateral apex narrow; apex complex, directed mesad, with apicomesal lobe-like processes, mesal process with sclerotized apex; dorsal branch absent. Phallus straight, narrow, with two spines and membranous pouch of eight small spines; phallotremal sclerite anterodorsal, large, hourglass-shaped.

***Holotype male:*** **ECUADOR:** Orellana, Reserva de Biodiversidad Tiputini, small stream, Harpia trail, 00.63496°S, 76.14602°W, el. 240 m, 22.x.2011, Holzenthal & Ríos [pinned] (UMSP000098911) (UMSP).

***Paratypes:*** same as holotype, except—four males [alcohol] (USFQ, MECN); same except: Reserva de Biodiversidade Tiputini, river slough, Numa trail, 00.63954°S, 76.14836°W, el. 260 m, 23.x.2011, Holzenthal & Ríos—one male [pinned] (UMSP).

**Etymology:** This new species in named for the Waorani people, in whose territory, now under severe environmental threat, this species occurs.

### Additional species records

**Table:** 

***Cernotina hastilis*** **Flint Jr, 1996, NEW RECORD**
Flint Jr, 1996:75 [original designation].—Botosaneanu, 2002:95 [checklist]. —Holzenthal & Calor, 2017:415 [catalog].

This species was previously recorded from the island of Tobago.

**Material examined:** **ECUADOR:** Orellana, Reserva de Biodiversidad Tiputini, small stream, Harpia trail, 00.63496°S, 76.14602°W, el. 240 m, 22. x.2011, Holzenthal and Ríos—two males [pinned] (UMSP); same except: 27 males [alcohol] (UMSP, MECN, USFQ).

**Table:** 

***Cernotinacygnea*** Flint Jr, 1971
*Cernotina cygnea* Flint Jr, 1971:37 [original description]. —Sykora, 1998:120 [distribution]. —Paprocki, Holzenthal & Blahnik, 2004:15 [checklist]. —Ríos-Touma et al., 2017:14 [distribution]. —Holzenthal & Calor, 2017:413 [catalog].

This species was previously reported from Brazil, Ecuador, and Peru.

**Material examined:** **ECUADOR:** Orellana, Reserva de Biodiversidad Tiputini, river slough, Numa trail, 00.63954°S, 76.14836°W, el. 260 m, 23. x. 2011, Holzenthal and Ríos—1 male [pinned] (UMSP).

**Table:** 

***Cernotinalobisomem*** **Santos & Nessimian, 2008**
*Cernotina lobisomem* Santos & Nessimian, 2008:27 [original description]. —Paprocki & França, 2014:82 [checklist]. —Ríos-Touma et al., 2017: 14 [distribution]. —Holzenthal & Calor, 2017:415 [catalog].

**Material examined:** **ECUADOR:** Orellana, Reserva de Biodiversidade Tiputini, river slough, Numa trail, 00.63954°S, 76.14836°W, el. 260 m, 23. x.2011, Holzenthal and Ríos—1 male [alcohol] (UMSP).

This species was previously reported from Brazil.

## Discussion

As discussed by Chamorro & Holzenthal (2010), the intermediate appendage in Polycentropodidae is difficult to distinguish in taxa where this structure is fused with tergum X along its mesal margin, a characteristic commonly found in *Cernotina*. This confusion has led to difficulty in determining the homology of the intermediate appendage versus the dorsolateral appendage in previous species descriptions (e.g., Holzenthal & Almeida, 2003). Some species such as *C*. *perpendicularis* Flint Jr, 1971 has an appendage very distinct from the membranous tergum X, similar to that of some *Polyplectropus*. In those cases, the intermediate appendages are lateral to tergum X, mesal to the dorsolateral process of the preanal appendages, and always setose.

In this paper, we used the term “intermediate appendage” to refer to the lateral, setose, lightly sclerotized lobes of tergum X, following the morphological discussions of Chamorro & Holzenthal (2010) for *Polyplectropus* and the character coding from Chamorro & Holzenthal (2011).

## Conclusions

The species of *Cernotina* described and recorded here were collected only adjacent to two small waterways, one a permanent small stream, the other an inundated, separated channel of the Tiputini River. We did not collect any specimen from lights set adjacent to the Tiputuni River. Even though the study consisted of only three nights of sampling (one on the Tiputini, two on the small water bodies), we collected five species, three recorded here and two species previously reported from Ecuador by Ríos-Touma et al. (2017). Considering the amount of similar freshwater habitats, the potential diversity of this genus in northern Amazonia is enormous. However, several species could become locally extinct due to the effects of environmental degradation from crude oil extraction, mining, and deforestation if current conservation efforts are not maintained. Loss of species diversity could be even greater, especially if regional endemism is also high as might occur with some *Cernotina* (Flint Jr, 1971). The importance of areas such as Tiputini and Yasuní cannot be overstated for the conservation of the largely unknown freshwater insect fauna of the Amazon.

##  Supplemental Information

10.7717/peerj.3960/supp-1 File S1 Distribution of new Ecuadorian *Cernotina* species, .kml file for Google Earth

## Additional Information and Declarations

### Field Study Permissions

The following information was supplied relating to field study approvals (i.e., approving body and any reference numbers): All collections were performed under the Environmental Ministry of Ecuador study permit No 0032 MAE-DPO-PNY-2011.

### Data Availability

The following information was supplied regarding data availability: The specimens examined in this work are deposited in the University of Minnesota Insect Collection, St. Paul, Minnesota, USA (UMSP); the Museo Ecuatoriano de Ciencias Naturales, Quito, Ecuador (MECN); and the Museo de Ecología Acuática de la Universidad San Francisco de Quito, Ecuador (USFQ).

### New Species Registration

The following information was supplied regarding the registration of a newly described species: Publication LSID: urn:lsid:zoobank.org:pub:5CE7AFEF-5077-4930-96BA-5B746FF12250. *Cernotina tiputini* LSID: urn:lsid:zoobank.org:act:E254D21B-7FA0-47CA-AF34-BE437CEE71CE. *Cernotina waorani* LSID: urn:lsid:zoobank.org:act:15FD59A3-69F2-4152-B7B8-EE5E34051603.
